# Molecular subtypes, tumor microenvironment infiltration characterization and prognosis model based on cuproptosis in bladder cancer

**DOI:** 10.7717/peerj.15088

**Published:** 2023-04-06

**Authors:** Heping Zhang, Ji Liu, Zongtai Zheng

**Affiliations:** 1 Department of Oncology, Shanghai Tenth People’s Hospital, School of Medicine, Tongji University, Shanghai, China; 2 Department of Urology, Shanghai Tenth People’s Hospital, School of Medicine, Tongji University, Shanghai, China; 3 Department of Urology, Guangdong Second Provincial General Hospital, Guangzhou, China

**Keywords:** Cuproptosis, Bladder cancer, Tumor microenvironment, Molecular subtypes

## Abstract

Cuproptosis is a kind of cell death dependent on copper. We aimed to explore the functions of the cuproptosis in the tumor microenvironment (TME) and construct a cuproptosis-related prognosis signature in bladder cancer (BCa). Using BCa patients in the public cohort, the cuproptosis-related molecular subtypes and cuproptosis-related prognosis signature were developed. Three cuproptosis-related molecular subtypes, with different prognoses and TME characteristics, were identified in BCa. The cuproptosis-related prognosis signature can divide patients into high- and low-risk groups with different prognoses, TME characteristics, chemotherapeutic drug susceptibility and immunotherapeutic response. Low risk group patients had a favored prognosis and response to immunotherapy. The dysregulation of cuproptosis-related genes expression levels was validated in multiple BCa cells using *in vitro* experiments. Cuproptosis has an important role in the tumor progression and the characterization of TME in BCa. The cuproptosis-related prognosis signature is a useful biomarker that can reflect the prognosis, TME characteristics, immunotherapeutic response and chemotherapeutic drug susceptibility in BCa patients.

## Introduction

Bladder cancer (BCa) is an aggressive cancer characterized by high rates of recurrence and metastasis (Babjuk et al., 2022; Witjes et al., 2021). The 2020 Global Cancer Statistics has reported that there were 573,278 new BCa patients worldwide, accounting for 3% of all newly diagnosed tumor patients; 212,536 deaths from BCa, accounting for 2.1% of all patients died of tumor (Sung et al., 2021) . Based on muscle-invasive status, BCa is broadly classified into nonmuscle invasive bladder cancer (NMIBC, 75%) and muscle invasive bladder cancer (MIBC, 25%) with distinct treatments and prognosis (Babjuk et al., 2022; Montironi et al., 2016; Witjes et al., 2021). Currently, targeted therapy, radiotherapy and immunotherapy for treating BCa patients, tumor recurrence and metastasis remain the main cause of treatment failure and fatal survival outcomes. Thus, identifying molecular subtypes of BCa is necessary to reduce the rates of recurrence and metastasis.

Based on gene expression, there are several types of molecular classifications, including TCGA (Robertson et al., 2018), Baylor (Mo et al., 2018), Lund (Lindgren et al., 2010), UNC (Damrauer et al., 2014), MDA (Choi et al., 2014), CIT (Rebouissou et al., 2014) and EUA (Kamoun et al., 2020).These molecular classifications can distinguish the biological characteristics and prognosis of BCa patients among molecular subtypes. However, the functions of these molecular classifications in treatment decision-making remain unidentified in BCa. Therefore, developing new molecular subtypes that can guide therapy need to be considered. Programmed cell death plays an important role in tumorigenesis, tumor progression and therapy (Labi & Erlacher, 2015; Lee et al., 2018; Li et al., 2019). Chemotherapeutic drugs that induce autophagy or apoptosis of cell death, like gemcitabine and cisplatin, have commonly been used in the treatment of BCa and achieved some clinical benefits (Goel et al., 2019; Lin et al., 2017). Cuproptosis occurs through the combination of copper with lipoylated components in the tricarboxylic acid cycle. This process promotes the loss of subsequentiron-sulfur cluster protein and the aggregation of the lipoylated protein, which leads to apoptosis (Tsvetkov et al., 2022). A tumor with a high concentration of lipoylated proteins is sensitive to cuproptosis, revealing that copper ionophore treatment may be an alternative treatment to these tumors that are resistant to traditional therapies.

We aimed to perform a comprehensive study to investigate the role of cuproptosis in BCa from a tumor microenvironment (TME) perspective. In this study, 1,306 BCa patients were classified into different cuproptosis-related subtypes, and the differences in prognosis and TME characteristics among the three subtypes were investigated. A cuproptosis prognosis signature was constructed to predict survival outcomes and characterize the chemotherapeutic drug susceptibility and the immune landscape of BCa. The results revealed that the cuproptosis score may be a useful prognostic marker. In addition, we further performed *in vitro* experiments to explore the expression pattern of cuproptosis genes and validate the performance of the cuproptosis-related prognosis signature.

## Materials and Methods

### Data collection

Figure S1 demonstrated the process of this study.

In Gene Expression Omnibus (GEO, https://www.ncbi.nlm.nih.gov/geo/), datasets were included based on the following criteria: (1) Histologically diagnosed as BCa; (2) Availability of RNA expression data; (3) Availability of prognostic information; (4) More than 50 tumor samples. In this way, the normalized gene expression data of five BCa datasets (GSE48277, GSE32894, GSE31684, GSE48075 and GSE13507) with relevant prognostic data were obtained from the GEO. The RNA-sequence data (fragments per kilobase million, FPKM) with clinical information of patients were obtained from a BCa dataset in The Cancer Genome Atlas (TCGA) (https://portal.gdc.cancer.gov). The gene expression data and corresponding clinicopathological and follow-up information of metastatic urothelial cancer patients who accepted atezolizumab (PD-L1 inhibitor) were obtained from the IMvigor210 trial (http://research-pub.gene.com/IMvigor210CoreBiologies). BCa patients in five GEO datasets and IMvigor210 trial were combined through the “Combat” method from the “sva” R package (https://doi.org/doi:10.18129/B9.bioc.sva) (Leek et al., 2012) to minimize the batch effects. “Combat” method could adjust data for batch effects through an empirical Bayes framework and return an expression matrix that has been corrected for batch effects. As a result, there were 1,306 BCa patients in the combined dataset Table S1 showed the main information on the combined dataset.

### Consensus clustering analysis of cuproptosis-related genes

Ten cuproptosis-related genes, including ferredoxin 1 (*FDX1*), lipoic acid synthetase (*LIAS*), lipoyltransferase 1 (*LIPT1*), dihydrolipoamide dehydrogenase (*DLD1*), dihydrolipoamide S-acetyltransferase (*DLAT*), pyruvate dehydrogenase E1 subunit alpha 1 (*PDHA1*), pyruvate dehydrogenase E1 subunit beta (*PDHB*), metal regulatory transcription factor 1 (*MTF1*), glutaminase (*GLS*) and cyclin dependent kinase inhibitor 2A (*CDKN2A*), were downloaded from a recent study (Tsvetkov et al., 2022). The network of genes biologically associated with cuproptosis-related genes was created in GeneMANIA (http://genemania.org/). Based on cuproptosis-related genes, BCa patients in the combined datasets were classified into distinct molecular subtypes using the R package “ConsensusClusterPlus”. The number of subtypes (K value) was determined according to three criteria: First, the sample sizes of each subtype were relatively equal. Second, the inter-subtype correlation decreased and the intra-subtype correlation increased after clustering. Third, the curve of the cumulative distribution function raised smoothly and gradually.

### Associations of molecular subtypes with clinicopathological features

The correlation of molecular subtypes with clinical features and prognoses was investigated. In addition, Kaplan–Meier and log-rank tests were performed using R packages “survminer” and “survival”.

### Evaluation of tumor-infiltrating immune cells (TIICs), Tumor immune dysfunction and exclusion (TIDE) and TME scores

The relative proportion of TIICs in each BCa patient was evaluated using CIBERSORT (https://cibersort.stanford.edu/) (Newman et al., 2015). The TIDE values of BCa patients were evaluated on the TIDE website (http://tide.dfci.harvard.edu/) (Fu et al., 2020). The ‘ESTIMATE’ R package was applicated to evaluate the TME scores (https://bioinformatics.mdanderson.org/estimate/index.html) (Yoshihara et al., 2013).

### Identification of differentially expressed genes (DEGs)

The DEGs among subtypes were obtained through the R package “limma” with a false discovery rate (FDR) <0.05 and log2—fold-change—>1.5. The potential functions of cuproptosis pattern-related DEGs were explored through the Gene Ontology (GO)/Kyoto Encyclopedia of Genes Genomes (KEGG) analyses in DAVID (https://david.ncifcrf.gov/). Gene Set Enrichment Analysis (GSEA) was performed using GSEA software (http://www.broad.mit.edu/GSEA/) to uncover biological processes based on sets of differentially expressed genes instead of individual genes. Gene Set Variation Analysis (GSVA) was performed using the R package “GSEABase” and “GSVA” to converts gene expression to pathways using gene sets. In GSEA and GSVA analysis, the KEGG (http://www.gsea-msigdb.org/gsea/msigdb/human/genesets.jsp?collection=CP:KEGG) and GO (http://www.gsea-msigdb.org/gsea/msigdb/human/genesets.jsp?collection=GO) gene sets were used. In addition, the cuproptosis score was calculated to quantitatively evaluate the enrichment scores of cuproptosis based on the gene expression of cuproptosis-related genes using the GSVA method.

### Cuproptosis gene cluster and cuproptosis-related prognosis signature

We further tried to construct the cuproptosis gene cluster and a cuproptosis-related prognosis signature. First, the univariate Cox regression and Kaplan–Meier analysis were performed on DEGs to obtain the prognostic DEGs. Second, Patients were divided into distinct cuproptosis gene clusters according to the gene expression of the prognostic DEGs using the R package “ConsensusClusterPlus”. Finally prognostic DEGs were introduced into the least absolute shrinkage and selection operator (LASSO) model was used to develop the cuproptosis-related prognosis signature *via* the R package “glmnet”, and the penalty regularization parameter lambda (*λ*) was chosen using 10-fold cross validation to obtain genes with non-zero coefficients and minimize the mean square error. Meanwhile, the minimal *λ* was identified to obtain the genes. The risk scores of the cuproptosis-related prognosis signature were calculated as follows: cuproptosis-related prognosis signature = Coef_1_ × expression of gene 1 + Coef_2_ × expression of gene 2 + …+ Coef_m_ × expression of gene m. Coef denotes the corresponding coefficient of the gene.

### Chemotherapeutic drugs susceptibility analysis and mutation

The semi-inhibitory concentration (IC50) of BCa patients was evaluated based on the R package “pRRophetic”. The somatic mutations of BCa patients in TCGA were demonstrated by the R package “maftools”.

### Development of the nomogram

The cuproptosis-related prognosis signature and clinical factors were subjected to univariate cox analysis. Factors significant in univariate cox analysis were then subjected to multivariate cox analysis to develop a nomogram. Decision curve analysis (DCA), Time-dependent receiver operating characteristic curve (ROC) curves and Calibration plots were applied to explore the performance of the nomogram and clinical factors.

### Cell culture and Real-time quantitative PCR (RT-qPCR)

All cell experiments were approved by the Ethics Committee of Shanghai Tenth People’s Hospital (approval number 2021KN108).

BCa cell lines (5637, T24, RT4, J82, and UMUC3) and human normal bladder epithelial cell line SV-HUC-1 were all purchased from the Chinese Academy of Sciences. RT4 was cultured in McCoy’s 5A medium (Thermo Fisher Scientific, Waltham, MA, USA); SV-HUC-1 was cultured in F12k medium (Sigma-Aldrich, St. Louis, MO, USA); J82 was cultured in DMEM (Thermo Fisher Scientific); T24, 5637 and UMUC3 were cultured in RPMI-1640 medium (Thermo Fisher Scientific). All cells were cultured at 37 °C and 5% CO2. All cell culture medias were supplemented with 10% fetal bovine serum (Gibco, Waltham, MA, USA). The protocols of total RNA extraction and RT-qPCR have been described previously (Wang et al., 2021). Data were analyzed with Bio-Rad software (Bio-Rad, Hercules, California, USA). Data were normalized by *β-actin* expression level in each sample. 2^−ΔΔCt^ method was used to calculate relative expression changes (Livak & Schmittgen, 2001). Primer sequences are presented in Table S2.

### Statistics

Principal component analysis (PCA) was conducted using the R package “pcaMethods”. The correlations among factors were investigated using the R package “corrplot”, “circlize”, “igraph” and “reshape2”. The cutoff points of the cuproptosis score and risk score were determined by the R package “survminer”. SPSS 23.0 (IBM, NY, USA) and R v.4.0.3 software (R Core Team, 2020) and were used for statistical analysis. Variables between subtypes or groups were compared using Mann–Whitney *U* test, Chi-square test, or Student’s *t*-test, as appropriate. A *P* < 0.05 in two-tailed analyses was statistical significance, with a Bonferroni correction applied for pairwise comparisons.

## Results

### Identification of cuproptosis subtypes in BCa

**Figure 1:**
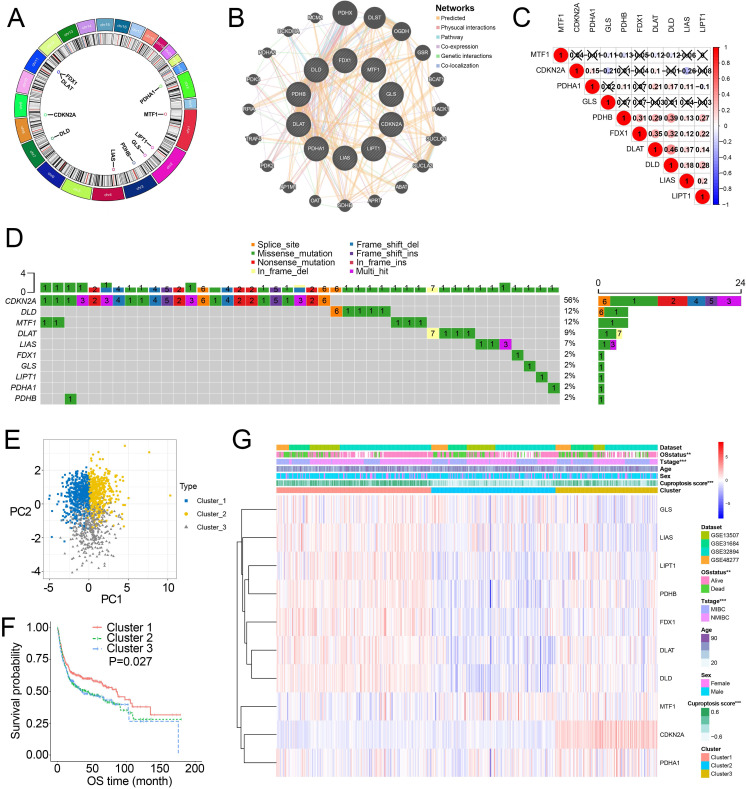
Landscape of cuproptosis-related genes and biological characteristics of cuproptosis-related molecular subtypes in BCa. (A) Locations of cuproptosis-related genes on 23 chromosomes. (B) Interaction of cuproptosis-related genes. (C) Correlation analysis between cuproptosis-related genes. Numbers in the square represent correlation coefficients. Crosses in the square represent *P* > 0.05. (D) Mutation frequencies of cuproptosis-related genes in BCa patients in TCGA. (E) Principal component analysis of cuproptosis-related genes in the combined dataset identified three distinct subtypes. (F) Kaplan–Meier curves for overall survival of combined dataset with the cuproptosis-related subtypes. (G) Differences in clinicopathologic features between three distinct subtypes. BCa, bladder cancer; OS, overall survival; NMIBC, nonmuscle invasive bladder cancer; MIBC, muscle invasive bladder cancer; TCGA, the Cancer Genome Atlas. *, *p* < 0.01, ∗∗∗, *p* < 0.001. *p* value was calculated with *t* test, except survival analysis was analyzed using a two-sided log-rank test.

Figure 1A showed the chromosome locations of the cuproptosis-related genes. GeneMANIA network built around cuproptosis-related genes demonstrated the top 20 genes interacted with cuproptosis-related genes (Fig. 1B). High interactions within cuproptosis-related genes were also showed in this network. The correlation network among cuproptosis-related genes were shown in Fig. 1C. Then, we investigated somatic mutations in cuproptosis-related genes in TCGA and discovered relatively low somatic mutations in these genes (Fig. 1D). Among them, *CDKN2A* had the highest somatic mutations while others had five or less than five somatic mutations.

Through the “Combat” algorithm, the batch effects were eliminated in the combined dataset (including GSE13507, GSE31684, GSE32894, GSE48075, GSE48277 and IMvigor210 trial) (Figs. S2A, S2B). The gene expression data of cuproptosis-related genes were used to categorize the BCa patients in the combined dataset. As a result, *K* = 3 was an optimal value to classify the BCa patients in the combined dataset into Cluster 1/2/3 (*n* = 515/427/364) (Figs. S2C–S2E). PCA plot showed novel differences among the three clusters (Fig. 1E). Survival analysis revealed a poorer overall survival (OS) in Cluster 2/3 patients than those in Cluster 1 patients (*P* = 0.027; Fig. 1F, Fig. S2F). Ten cuproptosis-related genes had significantly different expression patterns among these three clusters (Fig. 1G, Fig. S2G). In addition, Cluster 2 and Cluster 3 were preferentially related to the high T stage and higher risk of death (Fig. 1H).

### Characteristics of cuproptosis score and TIICs infiltration in the cuproptosis subtypes

**Figure 2:**
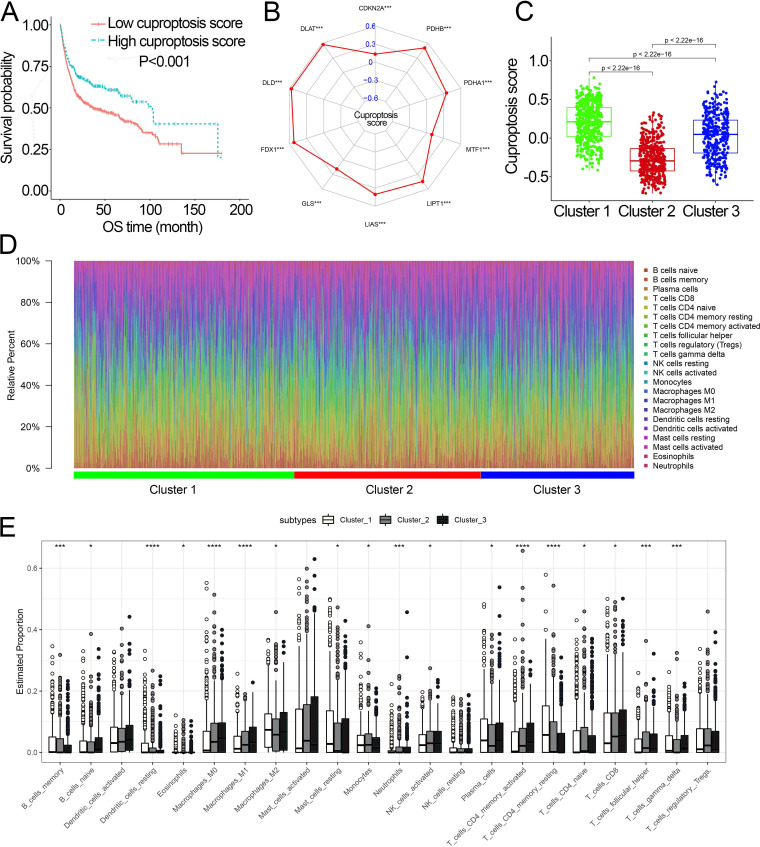
The association of the cuproptosis-related molecular subtypes with cuproptosis score and TIICs. (A) Kaplan–Meier curves of the cuproptosis score in the combined dataset. (B) The association of the cuproptosis score with cuproptosis-related genes. *p* value was calculated with Pearson correlation. (C) The different levels of cuproptosis scores among cuproptosis-related molecular subtypes. (D) The relative infiltration percentage of 22 TIICs of each BCa patient in the combined dataset. (E) The relative infiltration levels of TIICs based on the cuproptosis-related molecular subtypes. TIICs, tumor-infiltrating immune cells; OS, overall survival; BCa, bladder cancer. *, *p* < 0.05; **, *p* < 0.01; ***, *p* < 0.001; ****, *p* < 0.0001. *p* value was calculated with *t* test, Bonferroni correction applied for pairwise comparisons. Survival analysis was analyzed using a two-sided log-rank test.

Survival analysis showed that cuproptosis score was a protective biomarker in BCa patients (Fig. 2A). We then explored the correlation of the cuproptosis score with ten cuproptosis-related genes and cuproptosis subtypes. The results revealed that ten cuproptosis-related genes all had a positive correlation with the cuproptosis score (Fig. 2B, Fig. S3). Cluster 1 patients had the highest cuproptosis scores while Cluster 2 patients had the lowest cuproptosis scores (Fig. 2C).

We further investigated the differences in the proportion of TIICs among cuproptosis subtypes. The relative proportion of the 22 TIICs in each BCa patient was calculated and showed in Figs. 2D, 2E. Eighteen of 22 TIICs had significantly differently composition among cuproptosis subtypes (Fig. 2E), which revealed the diverse tumor immune cell microenvironments among cuproptosis subtypes.

### Identification of DEGs and cuproptosis gene cluster

**Figure 3:**
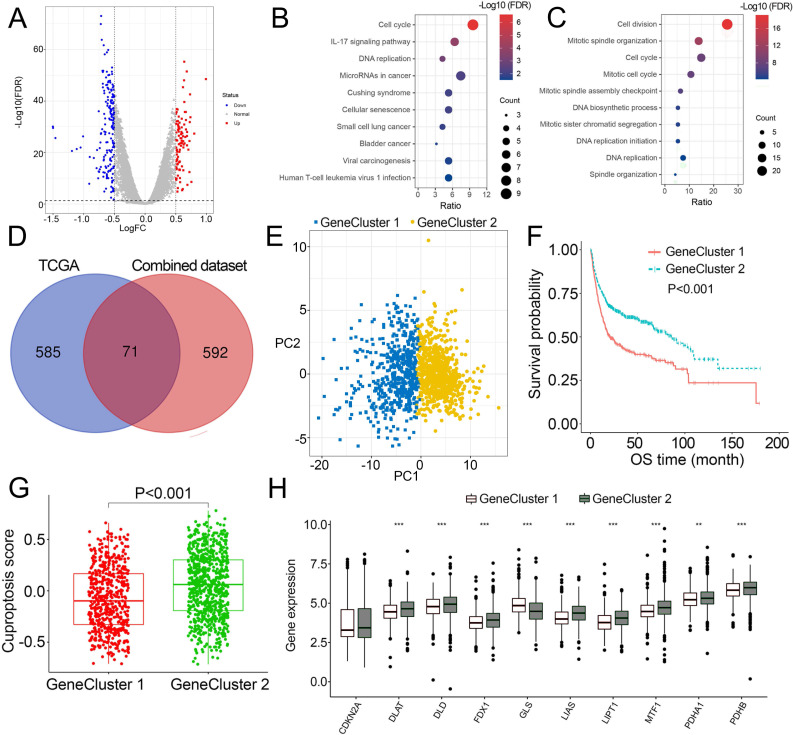
Landscape of biological characteristics of cuproptosis gene cluster. (A) Volcano plot of DEGs differentially expressed between Cluster 1 and Cluster 2/3. (B–C) KEGG and GO enrichment analyses of up-regulated DEGs in Cluster 2/3. (D) Venn diagram of the overlapping prognostic DEGs between TCGA and combined dataset. (E) Principal component analysis of overlapping prognostic DEGs in the combined dataset identified two distinct subtypes. (F) Kaplan–Meier curves of the cuproptosis gene cluster in the combined dataset. (G) The different levels of cuproptosis scores between GeneCluster 1 and GeneCluster 2. (H) Expression of cuproptosis-related genes between GeneCluster 1 and GeneCluster 2. TCGA, the Cancer Genome Atlas; FDR, false discovery rate; DEGs, differentially expressed genes; KEGG, Kyoto Encyclopedia of Genes Genomes; GO, Gene Ontology; OS, overall survival. *, *p* < 0.05, **, *p* < 0.01, ***, *p* < 0.001. *p* value was calculated with *t* test, except survival analysis was analyzed using a two-sided log-rank test.

As patients in Cluster 2/3 had similar survival outcomes, we combined Cluster 2 and Cluster 3 (named Cluster 2/3) and identified cuproptosis subtype-related DEGs between Cluster 2/3 and Cluster 1. In this way, 291 cuproptosis subtype-related DEGs were obtained (Fig. 3A), and DEGs that were up-regulated in Cluster 2/3 were enriched in many cancer-promoting biological processes (Figs. 3B, 3C), including Cell division, Cell cycle, DNA biosynthetic process, DNA replication, DNA replication initiation. The cuproptosis subtype-related DEGs were then introduced to univariate Cox analysis and Kaplan–Meier analysis in TCGA and combined dataset to obtain the prognostic DEGs. In this way, 71 prognostic DEGs were the overlapping genes related to OS in TCGA and combined data (Fig. 3D, Table S3). Based on the above genes, BCa patients in the combined dataset were divided into two genomic subtypes using the consensus clustering algorithm, namely GeneCluster 1 and GeneCluster 2 (Figs. S4A–S4F). PCA plot showed significant differences in the cuproptosis transcription profiles among two cuproptosis gene subtypes (Fig. 3E). Patients in GeneCluster 2 had significantly poorer OS than those in GeneCluster 1 (*P* < 0.001, Fig. 3F), and BCa patients in GeneCluster 2 had significantly higher cuproptosis scores compared with BCa patients in GeneCluster 1 (Fig. 3G). In addition, there were significant differences in clinicopathological features (Fig. S4G), TME characteristics (Figs. S5A, S5B) and cuproptosis-related gene expression (Fig. 3H) between GeneCluster 1 and GeneCluster 2.

### Development of the cuproptosis-related prognosis signature

**Figure 4:**
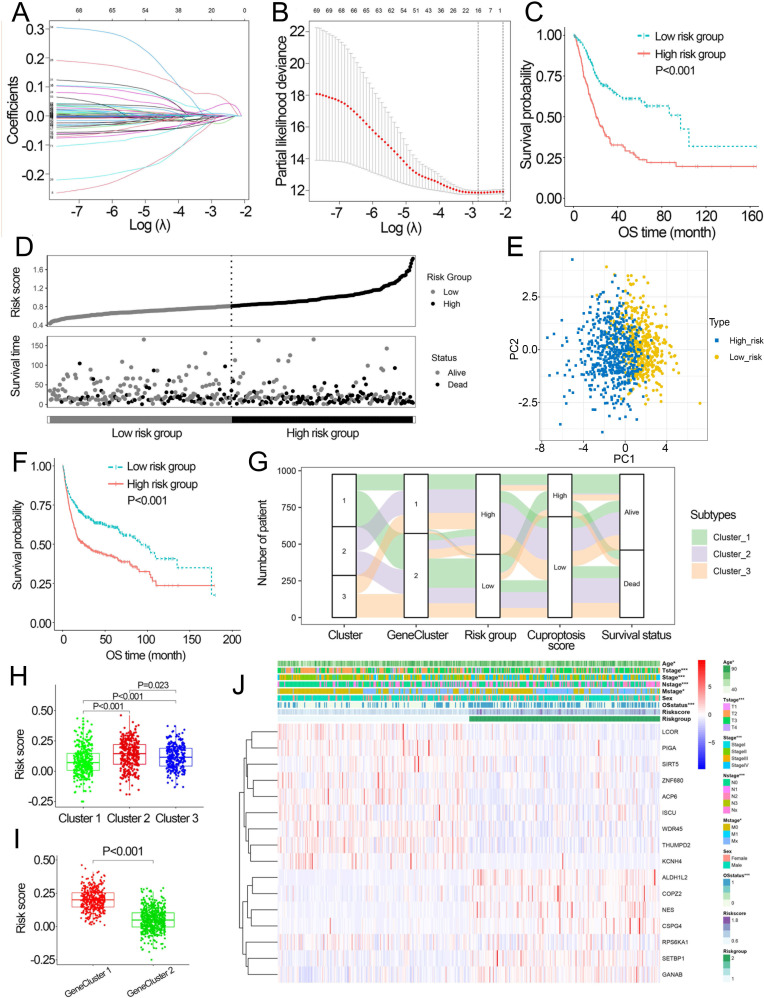
Development and performance of the cuproptosis-related prognosis signature. (A) Based on the optimal *λ* value (−2.840), 16 genes were selected. (B) Selecting the optimal number of genes based on minimum partial likelihood deviance in TCGA. (C) Kaplan–Meier curves of the cuproptosis-related prognosis signature in TCGA. (D) Ranked dot and scatter plots showing the risk scores distribution and patient survival status. (E) Principal component analysis of 16 genes in TCGA identified two risk groups. (F) Kaplan–Meier curves of the cuproptosis-related prognosis signature in the combined dataset. (G) Alluvial diagram showing the changes of cuproptosis-related molecular subtype, gene cluster, risk group, cuproptosis score group and survival status. (H) The different levels of risk scores among cuproptosis-related molecular subtypes. (I) The different levels of risk scores between GeneCluster 1 and GeneCluster 2. (J) Differences in clinicopathologic features between two risk groups. TCGA, the Cancer Genome Atlas; OS, overall survival. *p* value was calculated with *t* test, Bonferroni correction applied for pairwise comparisons. Survival analysis was analyzed using a two-sided log-rank test.

Based on the minimal *λ* (−2.840), 16 hub genes with their corresponding coefficients were obtained among prognostic DEGs to develop the cuproptosis-related prognosis signature (Fig. 4A, Fig. S6A), in which the partial likelihood deviance was at its minimum (Fig. 4B). The correlations of 16 hub genes in the cuproptosis-related prognosis signature were demonstrated in Fig. S6B, suggesting that these genes were not highly correlated with each other. The risk scores were calculated by summing the genes weighted by corresponding coefficients, which was mentioned above. The optimal cutoff point for risk scores was 0.812 determined by the R package “survminer” in TCGA. BCa patients were classified into high- and low-risk groups according to the optimal cutoff point, and high-risk patients had significantly lower survival rates and poorer OS than low-risk patients (Figs. 4C, 4D). Based on the prognostic DEGs, PCA was performed and revealed that BCa patients in low or high risk groups were distributed in discrete directions indicating novel differences in the prognostic DEGs between the high- and low-risk patients (Fig. 4E). To validate the prognostic value of the cuproptosis-related prognosis signature, BCa patients in the combined dataset were also divided into low- and high-risk groups. Kaplan–Meier curve showed that patients in high risk group also had poor OS than those in the low risk group in the combined dataset (Fig. 4F). The alluvial diagram illustrated the distribution of BCa patients in three cuproptosis subtypes, two risk groups, two gene subtypes, two cuproptosis score groups and survival outcomes (Fig. 4G). Patients in Cluster 2/3 and GeneCluster 1 had higher risk scores compared with those in Cluster 1 and GeneCluster 2, respectively (Figs. 4H, 4I). In TCGA, high-risk patients were preferentially correlated with higher age, higher TNM stage and low survival rate (Fig. 4J, Figs. S6C–S6G).

### Characteristics of the signature

**Figure 5:**
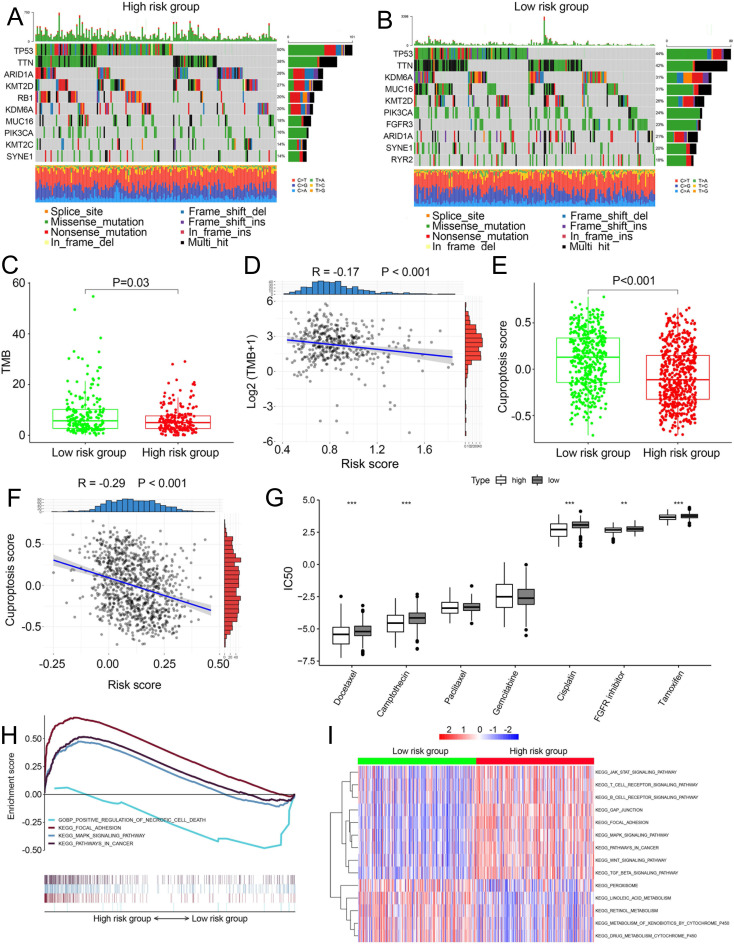
Landscape of biological characteristics of the cuproptosis-related prognosis signature (risk score). (A) The mutation frequency of high risk group. (B) The mutation frequency of low risk group. (C) The different levels of TME between low- and high-risk groups. (D) Correlations between risk score and TMB. (E) The different levels of cuproptosis score between low- and high-risk groups. (F) Correlations between risk score and cuproptosis score. *p* value and correlation coefficient R were calculated with Pearson correlation. (G) The different IC50 values of chemotherapeutic drugs between low and high risk groups. (H) GSEA analysis of the cuproptosis-related prognosis signature. (I) GSVA analyzed the different biological pathways between low- and high-risk groups. TMB, tumor mutation burden; IC50, semi-inhibitory concentration, GSEA: Gene Set Enrichment Analysis; GSVA, Gene Set Variation Analysis. *, *p* < 0.05, **, *p* < 0.01, ***, *p* < 0.001. *p* value was calculated with *t* test.

We compared somatic mutations, TMB, cuproptosis score and IC50 values of chemotherapeutic drugs between low- and high-risk patients. In TCGA, the top ten mutated genes were demonstrated in Figs. 5A and 5B, respectively. High-risk patients had lower TMB than those in low risk patients (*P* = 0.03, Fig. 5C). There was a negative correlation between TMB and risk scores (Fig. 5D, R =−0.17, *P* < 0.001). Similarly, in the combined dataset, low risk patients had higher cuproptosis score compared with high-risk patients (*P* < 0.001, Fig. 5E), and risk score was negatively related to the cuproptosis score (Fig. 5F). In addition, among seven common chemotherapeutic drugs used in BCa, the IC50 of five drugs were significantly different between two risk groups (Fig. 5G). GSEA revealed that several cancer-promoting pathways were highly enriched in high risk patient, including ‘Focal adhesion’, ‘MAPK signaling pathway’ and ‘Pathway in cancer’, while the ‘Positive regulation of necrotic cell death’ was enriched in low risk BCa patients (Fig. 5H). GSVA showed similar results with the ‘MAPK signaling pathway’, ‘JAK STAT signaling pathway’, ‘Focal adhesion’ and ‘Pathway in cancer’ and ‘WNT signaling pathway’ enriched in high risk BCa patients (Fig. 5I).

### Characteristics of the TME in distinct risk groups

**Figure 6:**
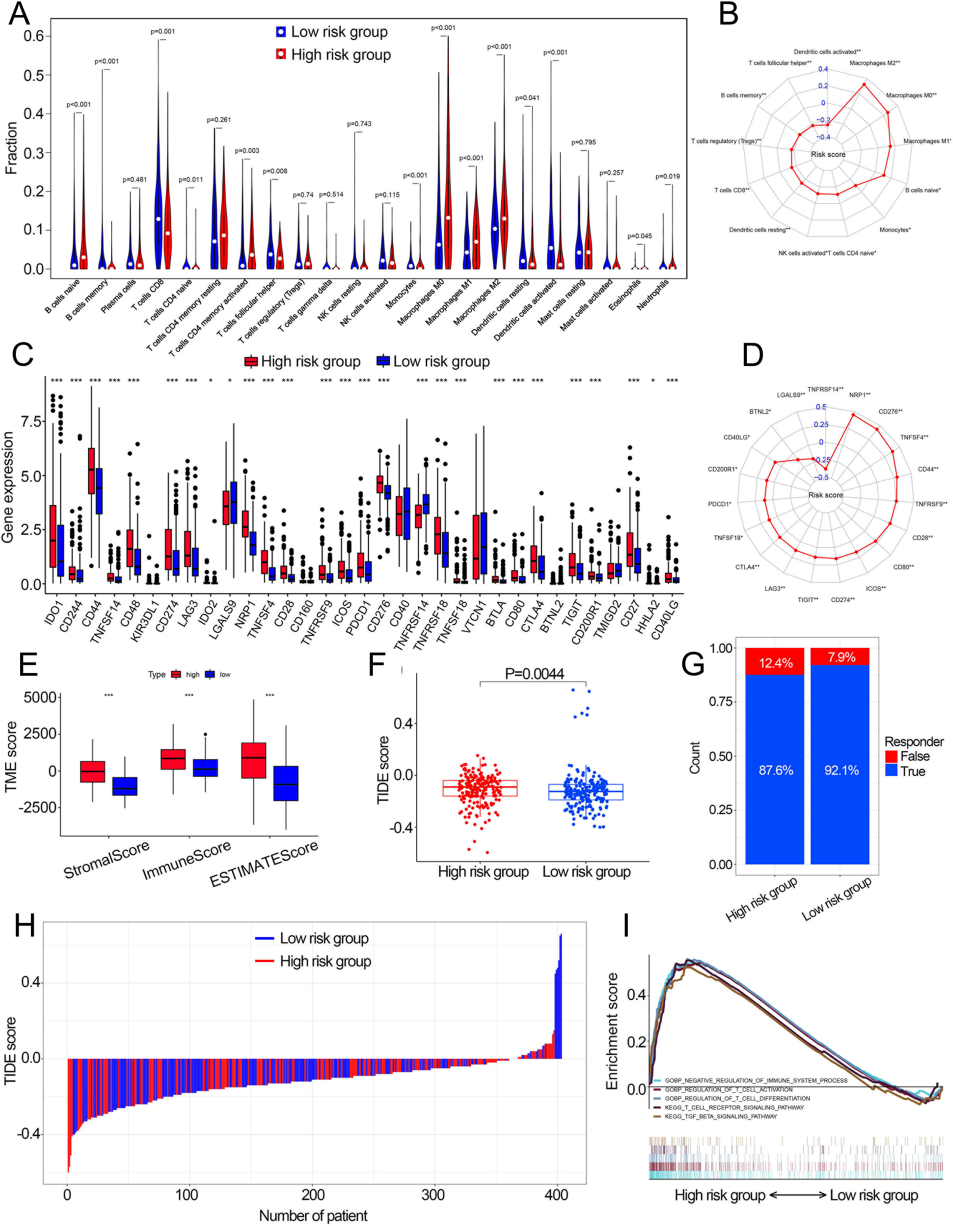
The correlation between the cuproptosis-related prognosis signature (risk score) and TME characteristics. (A) The relative infiltration levels of TIICs based on the risk groups. (B) The association of the risk score with the infiltration level of TIICs. *p* value was calculated with Pearson correlation. (C) The expression of immune checkpoints based on the risk groups. (D) The association of the risk score with the expression of immune checkpoints. *p* value was calculated with Pearson correlation. (E) The different levels of TME score between low- and high-risk groups. (F) The different levels of TIDE score between low- and high-risk groups. (G) Proportions of immunotherapy response in high- and low-risk groups. *p* value was calculated with Chi-square test. (H) The TIDE value of each BCa patient based on low- and high-risk groups. (I) GSEA analysis of the cuproptosis-related prognosis signature. TME, tumor microenvironment; TIICs, tumor-infiltrating immune cells; TIDE, Tumor immune dysfunction and exclusion; GSEA, Gene Set Enrichment Analysis. *, *p* < 0.05, **, *p* < 0.01, ***, *p* < 0.001. *p* value was calculated with *t* test except Chi-square test in (G).

Fourteen of 22 TIICs had different compositions between two risk groups (Fig. 6A, Table S4), which revealed the diverse tumor immune cell microenvironments between risk groups. Specifically, the proportions of macrophages were higher in high-risk patients compared with low risk patients, while T cells follicular helper, T cells CD8, B cells memory and monocytes contained significantly lower infiltration levels in high-risk patients compared to those in low risk patients. In addition, the risk score had a significant correlation with 12 TIICs (Fig. 6B, Fig. S7). We then investigated the relationship between the expression of some immune checkpoints and the risk score. The results revealed that some immune checkpoints had different expression levels between two risk groups, including *CD274*, *PDCD1* and *CTLA4* (Fig. 6C), and risk score had a correlation with some immune checkpoints (Fig. 6D). High-risk patients had higher immune scores, stromal scores and TIDE scores compared with low-risk patients (Figs. 6E, 6F). Based on the TIDE scores, high-risk patients had a lower proportion of responders compared to those in low-risk patients if received immunotherapy (Figs. 6G, 6H). These results indicated that this model could be a useful predictor of the efficacy of immunotherapy. GSEA and GSVA revealed that high-risk patients had multiple immune-related pathways enriched, including ‘Regulation of T cell activation’, ‘Negative regulation of immune system process’, ‘Regulation of T cell differentiation’, ‘T cell receptor signaling pathway’ and ‘TGF beta signaling pathway’ (Figs. 5I, 6I).

### Construction of the nomogram

**Figure 7:**
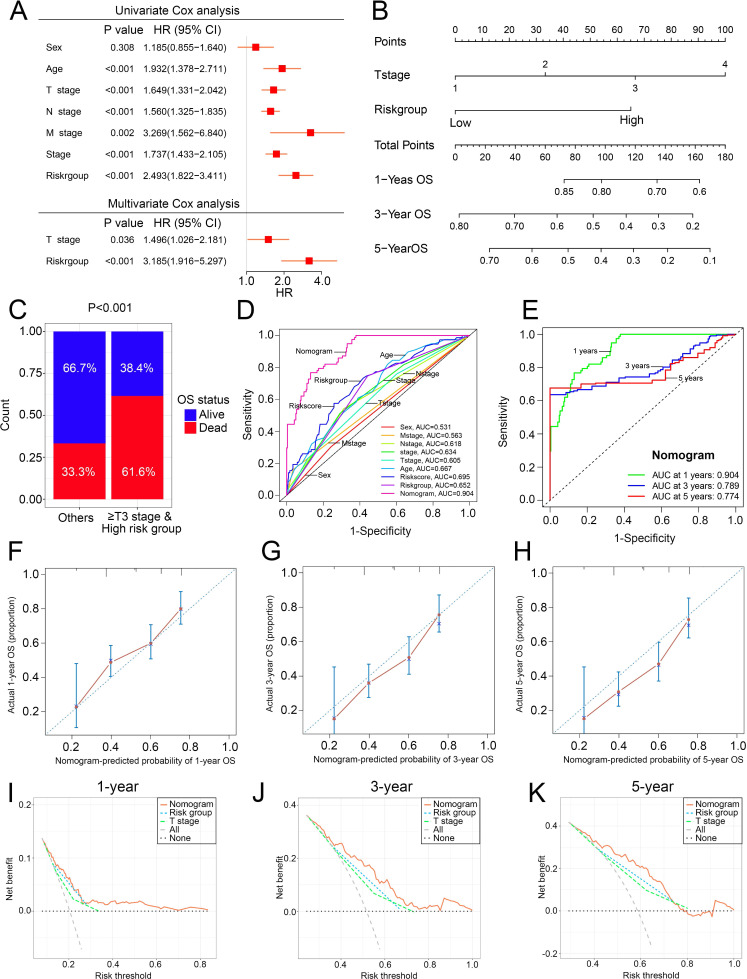
The construction and performance of the nomogram in TCGA. (A) Univariate and multivariate Cox analyses of the cuproptosis-related prognosis signature and other clinicopathological features. (B) Nomogram for predicting the probability of 1-, 3-, and 5-year OS. (C) Proportions of survival status in BCa patients ≥ T2 stage & high risk group and other patients. (D) ROC curves of the cuproptosis-related prognosis signature and other clinicopathological features for prognostic prediction. e ROC curves to predict the 1-, 3- and 5-year OS according to the nomogram. (F–H) Calibration plots of the nomogram for predicting the probability of 1-, 3-, and 5-year OS. (I–K) DCA of the nomogram predicting 1-, 3-, and 5-year OS. TCGA, the Cancer Genome Atlas; OS, overall survival; BCa, bladder cancer; ROC, receiver operating characteristic curve; DCA, decision curve analysis. *p* value was calculated with Chi-square test, except survival analysis was analyzed using univariate and multivariate Cox analysis.

To overcome the inconvenient clinical utility of the signature (risk score), a cuproptosis-related prognosis signature-based nomogram was constructed to predict the survival outcomes of BCa patients. The results of univariate and multivariate Cox regression analysis showed that risk score and T stage remained the independent prognostic factors (Fig. 7A), so the signature and T stage were selected to develop the nomogram (Fig. 7B). The histogram also showed that patients with T stage ≥T3 and high-risk patients had higher death rates than others (*P* < 0.001, Fig. 7C). The nomogram achieved the optimal performance in the prediction of patients’ survival outcomes compared with other factors (Figs. 7D–7H). Furthermore, the nomogram achieved higher clinical net benefit than the T stage and risk group (Figs. 7I–7K).

### The experimental validation of the cuproptosis-related genes

**Figure 8:**
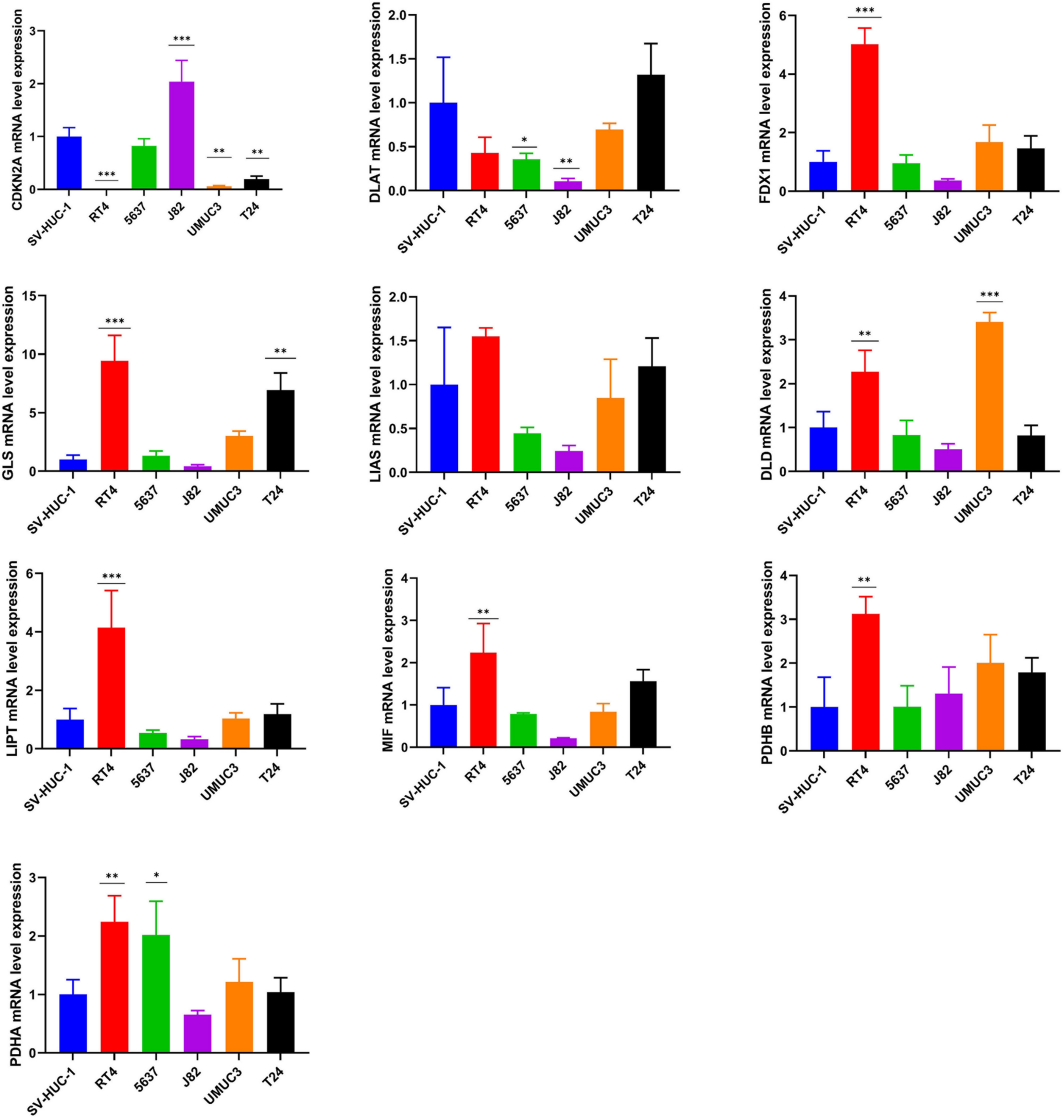
Validation of cuproptosis-related genes through *in vitro* experiments. *, *p* < 0.05; **, *p* < 0.01; ***, *p* < 0.001, *p* value was calculated with *t* test, compared to control group (SV- HUC-1). The data represent the mean ±s.e.m of three replicate experiments.

The mRNA expression pattern of the ten cuproptosis-related genes in five human BCa cell lines (T24, 5637, RT4, J82, and UMUC3) and human normal bladder epithelial cell line SV-HUC-1 was determined using RT-qPCR. The results showed significant differences in the expression levels of nine cuproptosis-related genes (except *LIAS*) between BCa and normal bladder epithelial cells (Fig. 8).

## Discussion

The heterogeneity of BCa leads to highly variable survival outcomes and makes antitumor treatment a demanding task. It is reported that the response rates to neoadjuvant chemotherapy were less than 50% in BCa (Grossman et al., 2003; Kitamura et al., 2014; Zargar et al., 2015), and the response rates to immunotherapy were only about 25% in BCa. (Bellmunt et al., 2017; Sharma et al., 2017; van der Heijden et al., 2021). Thus, it is necessary to accurately identify the subtypes of BCa for personalized therapy. Previous studies have developed various molecular classifications of BCa (Kamoun et al., 2020; Robertson et al., 2018; Sjödahl et al., 2012), but the molecular subtypes of these molecular classifications still cannot be used to determine the treatment strategy due to the considerable heterogeneity among the subtypes. Therefore, a more accurate molecular subtype of BCa is needed to guide personalized therapy and improve survival outcomes. Cuproptosis is a newly discovered cell death, and it depends on the combination of copper with lipoylated TCA cycle proteins (Tsvetkov et al., 2022). As copper is one of the cofactors for essential enzymes in human cells (Kim, Nevitt & Thiele, 2008), the identification of molecular subtypes based on cuproptosis may be a useful direction for clinical treatment.

To understand the significant role of cuproptosis in BCa, our study established three subtypes in BCa patients based on ten cuproptosis-related genes. Significant differences in prognoses and biological characteristics were found among the three subtypes. Several cancer-promoting biological pathways were highly enriched in Cluster 2/3, which partially explained the poor survival outcomes in Cluster 2/3. The proportions of the TIICs also differed significantly among the three subtypes. To investigate the functions of cuproptosis in BCa patients’ prognosis, we used the GSVA algorithm to create a biomarker named cuproptosis score according to ten cuproptosis-related genes. The results revealed that a high cuproptosis score was correlated with a favored prognosis, and BCa patients in Cluster 1 characterized by a favored prognosis have the highest cuproptosis score among the three subtypes. In addition, two gene subtypes (GeneCluster 1 and GeneCluster 2) were obtained based on the prognostic DEGs among the three subtypes. Significant differences in the RNA expression levels of most cuproptosis-related genes were found between GeneCluster 1 and GeneCluster 2, and BCa patients in GeneCluster 1 have significantly lower cuproptosis scores and poor OS. These findings indicated that cuproptosis has an important role in the tumor progression, TME and prognosis in BCa, and BCa patients with different cuproptosis patterns had different TME characteristics and prognoses.

Many studies developed prognosis signatures to predict the prognoses of tumor patients based on various kinds of cell death, including pyroptosis and ferroptosis (Song et al., 2021; Sun et al., 2021; Zhang et al., 2021). However, most of these prognosis signatures are based on single dataset without external validation or experimental validation, which limits the reproducibility and generalizability of the prognosis signatures. We aimed to established a useful prognosis signature and validated its prognostic value in other datasets. In addition, experimental validation was performed which could be lacked in other studies. In this study, a cuproptosis-related prognosis signature based on 16 prognostic genes was developed. As predicted, the cuproptosis-related prognosis signature was related to patients’ prognoses, high risk score was related to advanced TNM stage and poor survival outcomes. BCa patients in Cluster 1 and GeneCluster 2 characterized by favored prognosis have significantly lower risk scores compared to Cluster 2/3 and GeneCluster 1, respectively. The prognostic value of the cuproptosis-related prognosis signature was further explored in the combined dataset for external validation. As predicted, the cuproptosis-related prognosis signature still had a novel performance in survival prediction in the combined dataset, indicating that the cuproptosis-related prognosis signature may be a novel prognostic biomarker. Additionally, patients in low risk group have significantly higher IC50 values of five chemotherapeutic drugs compared to those in high-risk patients, which revealed that the cuproptosis-related prognosis signature may predict the therapeutic response of chemotherapeutic drugs and high-risk patients may benefit from chemotherapy. GSEA and GSVA further showed that there were several cancer-promoting pathways highly enriched in high-risk patients and a pathway of ‘positive regulation of necrotic cell death’ enriched in low-risk patients, revealing that two risk groups divided by the cuproptosis-related prognosis signature have distinct biological characteristics, thereby contributed to differences in prognosis and therapeutic response between two risk groups. The cuproptosis-related prognosis signature may be useful in survival prediction and personalized therapy in BCa.

While the use of chemotherapeutic drugs cannot produce a durable response and may lead to some serious side effects, immune checkpoint inhibitors (ICIs) is playing a vital role in the therapy of BCa patients. Clinical research has proved the safety of ICIs in human tumors (Rotte , 2019). For advanced and metastatic urothelial carcinoma in the post-platinum setting, several ICIs have been suggested as the second-line treatment (Bellmunt et al., 2017; Patel et al., 2018; Powles et al., 2017; Rosenberg et al., 2016; Sharma et al., 2017; van der Heijden et al., 2021). However, only about 25% BCa patients were sensitive to immunotherapy (Bellmunt et al., 2017; Sharma et al., 2017; van der Heijden et al., 2021), which highlight the necessary of constructing a reliable signature to identify subgroups of BCa patients benefit from ICIs. However, the specific mechanism between cuproptosis and TME is not clear. We then explored the function of cuproptosis in TME. Based on the cuproptosis-related prognosis signature, two risk groups had novel differences in the proportions of 14 TIICs and expression patterns of 27 immune checkpoints, and risk scores were highly related to most of the TIICs and immune checkpoints. Specifically, high-risk patients have a significantly higher abundance CD8+ T cells, B cells memory, CD4+ naive T cells and T-follicular helper cells, and low-risk patients have a significantly higher abundance of M2 macrophages and neutrophils. CD8+ T cells play an important role in tumor cell killing and therapeutic sensitivity of ICIs (Fridman et al., 2017; Powles et al., 2014), and patients with high abundance of tumor-infiltrating T cells have a favored prognosis in BCa (Sharma et al., 2007). Previous studies also showed that B cells in TME were correlated with better prognosis in BCa (Zirakzadeh et al., 2020), colorectal cancer (Berntsson et al., 2016) and sarcoma (Petitprez et al., 2020). In this study, T cells and B cells were highly enriched in the low-risk patients, which not only explained the good prognosis of low-risk patients but also revealed that low-risk patients possibly may benefit from immunotherapy. On the contrary, M2 macrophages can induce tumor immunosuppression and promote tumor progression through releasing proinflammatory cytokines including IL-10/12/13/16, INF- *γ* and transforming growth factor- *β* (Chen et al., 2019; Jeannin et al., 2018; Sica et al., 2006; Yunna et al., 2020), and tumor-associated neutrophil was another important member of TIICs and can release several factors leading to tumor progression (Eruslanov, 2017; Galdiero et al., 2013; Mantovani et al., 2011). Consistent with previous studies, an increased abundance of M2 macrophages and neutrophils was found in high risk group. High-risk patients have significantly higher TME scores than those in low-risk patients, which revealed that low-risk patients have lower relative infiltration of stromal cells and immunocytes in TME. As for the TIDE score, low-risk patients have higher responder rates and lower TIDE scores. TIDE was regarded as a surrogate biomarker that can predict response to ICIs, and a lower TIDE score is related to better ICIs response and better prognosis (Jiang et al., 2018). In our study, low TIDE scores contribute to the relatively high ICIs response and good prognosis in low-risk patients. GSEA further validated the dysfunction of the immune system in high-risk patients. These results illustrate that there were distinct immunophenotypes between low- and high-risk groups, and the cuproptosis-related prognosis signature is associated with the immunotherapy response. In this way, the cuproptosis-related prognosis signature may have the potential to facilitate individualized immunotherapy in BCa. As the cuproptosis-related prognosis signature could divided BCa patients into subgroups with different prognosis and immunophenotypes, we developed a nomogram combining the cuproptosis-related prognosis signature with the T stage to facilitate the integration of the cuproptosis-related prognosis signature into clinical practice. The ROC curves, Calibration plots and DCA revealed that the nomogram was a useful evaluation tool for prognostic predictions. This nomogram facilitated the use of the cuproptosis-related prognosis signature and improved the performance of survival prediction.

We further explore the mRNA expression pattern of the ten cuproptosis-related genes in human BCa cell lines using qPCR. The results revealed that most of cuproptosis-related genes exhibited dysregulated expression between a human normal bladder epithelial cell line and BCa cell lines, suggesting that dysregulated expression levels of cuproptosis-related genes served an important role in the carcinogenesis of BCa. Specifically, *CDKN2A* was downregulated in four BCa cell lines compared with the human normal bladder epithelial cell line. *CDKN2A* (also called p16) was an important tumor suppressor that prevents carcinogenesis through induction of senescence cell growth and arrest (Rayess, Wang & Srivatsan, 2012). *CDKN2A* promoter methylation is a frequent epigenetic event, which could result in silencing of *CDKN2A* expression and thereby leading to uncontrolled cell proliferation and carcinogenesis in several tumors (Riese et al., 1999; Samowitz et al., 2005; Saridaki et al., 2003) including BCa (Worst et al., 2018). However, the tumor suppression mechanism of *CDKN2A* from a cuproptosis perspective had not been investigated, which is worthy of studying.

This study has some limitations. First, additional experimental verification is needed to validate the relationship between TME and cuproptosis. Furthermore, some clinical factors including therapy were unavailable, which may lead to different cuprotosis state and TME.

In conclusion, we identified three subtypes of cuproptosis-associated molecules in BCa with distinct prognoses. Cuproptosis-related genes play important role in TME heterogeneity in BCa. The cuproptosis-related prognosis signature can distinguish the survival outcomes, biological characteristics, TIICs infiltration and immunotherapeutic response in BCa patients.

##  Supplemental Information

10.7717/peerj.15088/supp-1 Supplemental Information 1 Data set information included in this study for cuproptosis classification in the combined dataset

10.7717/peerj.15088/supp-2 Supplemental Information 2 Supplementary Table S2 qPCR primers

10.7717/peerj.15088/supp-3 Supplemental Information 3 Univariate Cox regression and Kaplan–Meier analysis of 71 cuproptosis related genes in TCGA

10.7717/peerj.15088/supp-4 Supplemental Information 4 The relative infiltration levels of TIICs based on the risk groups

10.7717/peerj.15088/supp-5 Supplemental Information 5 The entire analytical process of the study

10.7717/peerj.15088/supp-6 Supplemental Information 6 Elimination of the batch effects and unsupervised clustering of cuproptosis-related genes (A–B) PCA plots of the combined dataset before (A) and after (B) the elimination of the batch effects. c Consensus matrix heatmaps for *k* = 2. (D–E) Consensus clustering CDF (D) and relative change in area under CDF curve for *k* = 2–9 (E). (F) Kaplan–Meier curves for OS of the combined dataset with the cuproptosis-related subtypes. (G) The expression of cuproptosis-related genes among cuproptosis-related subtypes.

10.7717/peerj.15088/supp-7 Supplemental Information 7 The relationship between cuproptosis and expression level of cuproptosis-related genes in bladder cancer

10.7717/peerj.15088/supp-8 Supplemental Information 8 Development of the cuproptosis gene cluster in the combined dataset (A–D) Consensus matrix heatmaps for *k* = 2–5. (E–F) Consensus clustering CDF (E) and relative change in area under CDF curve for *k* = 2–9 (F). (G) Differences in clinicopathologic features between cuproptosis gene clusters.

10.7717/peerj.15088/supp-9 Supplemental Information 9 The correlation between the cuproptosis gene clusters and TME characteristics (A) The relative infiltration levels of TIICs based on the cuproptosis gene clusters. (B) The expression of immune checkpoints based on the cuproptosis gene clusters.

10.7717/peerj.15088/supp-10 Supplemental Information 10 The performance of the cuproptosis-related prognosis signature (risk score) (A) Coefficients of 16 genes in the cuproptosis-related prognosis signature. (B) Correlations among 16 genes in the cuproptosis-related prognosis signature. (C–G) Distribution of risk scores stratified by age (C), T stage (D), N stage (E), M stage (F) and Stage (G).

10.7717/peerj.15088/supp-11 Supplemental Information 11 The correlation between the cuproptosis-related prognosis signature (risk score) and tumor-infiltrating immune cells The correlation between the cuproptosis-related prognosis signature (risk score) and tumor-infiltrating immune cells.

10.7717/peerj.15088/supp-12 Supplemental Information 12 q-RCR raw data

10.7717/peerj.15088/supp-13 Supplemental Information 13 R Code of data combination

10.7717/peerj.15088/supp-14 Supplemental Information 14 R Code of Consensus clustering analysis

10.7717/peerj.15088/supp-15 Supplemental Information 15 R Code of LASSO

10.7717/peerj.15088/supp-16 Supplemental Information 16 R Code of vioplot

10.7717/peerj.15088/supp-17 Supplemental Information 17 R Code of correlation network

10.7717/peerj.15088/supp-18 Supplemental Information 18 R Code of correlation circos

## Additional Information and Declarations

### Data Availability

The following information was supplied regarding data availability: The code used in this study is available in the Supplementary File.
