# Supplementary material for: Molecular subtypes, tumor microenvironment infiltration characterization and prognosis model based on cuproptosis in bladder cancer
Source: PeerJ. 2023 Apr 6;11:e15088. doi: 10.7717/peerj.15088 (PMC10083007; doi:10.7717/peerj.15088)
Supplement: Supplemental Information 1 [file peerj-11-15088-s001.docx]

**Supplementary Table S1 Data set information included in this study for cuproptosis classification in the combined dataset**

| Accession number | Number of BCa patients | Number of LUAD patients with prognostic information | Platform | Method |
| --- | --- | --- | --- | --- |
| GSE13507 | 256 | 165 | GPL6102 | Illumina human-6 v2.0 expression beadchip |
| GSE31684 | 93 | 93 | GPL570 | Affymetrix Human Genome U133 Plus 2.0 Array |
| GSE32894 | 308 | 308 | GPL6947 | Illumina HumanHT-12 V3.0 expression beadchip |
| GSE48075 | 142 | 73 | GPL6947 | Illumina HumanHT-12 V3.0 expression beadchip |
| GSE48277 | 159 | 73 | GPL14951 | Illumina HumanHT-12 WG-DASL V4.0 R2 expression beadchip |
| IMvigor210C | 348 | 348 | - | TruSeq RNA Access technology |
