# Supplementary material for: Molecular subtypes, tumor microenvironment infiltration characterization and prognosis model based on cuproptosis in bladder cancer
Source: PeerJ. 2023 Apr 6;11:e15088. doi: 10.7717/peerj.15088 (PMC10083007; doi:10.7717/peerj.15088)
Supplement: Supplemental Information 2 [file peerj-11-15088-s002.docx]

**Supplementary Table S2 qPCR primers.**

| Type | Primers |
| --- | --- |
| *CDKN2A*: sense | 5′-GATGTCGCACGGTACCTG-3′ |
| *CDKN2A*: antisense | 5′-TCTCTGGTTCTTTCAATCGGG-3′ |
| *DLAT* :sense | 5′-CGGAACTCCACGAGTGACC-3′ |
| *DLAT* :antisense | 5′-CCCCGCCATACCCTGTAGT-3′ |
| *DLD* :sense | 5′-AACACCAACTCCACACTCTG-3′ |
| *DLD* :antisense | 5′-TTCCTTCTGCCCAAACTCTG-3′ |
| *FDX1*: sense | 5′-CTAACCCACACCTGAGATAACG-3′ |
| *FDX1*: antisense | 5′-TGAATGTCCCCTCCAAAACTC-3′ |
| *GLS*:sense | 5′--CATTACCTAGATGGCACCTCC--3′ |
| *GLS* :antisense | 5′-GTCTCCATGGCTTGCTAGATC-3′ |
| *LIAS* :sense | 5′-CAGCCCAGTCAGACCGTTAAG-3′ |
| *LIAS* :antisense | 5′-TTTCTGGCGTTTTAGGTTTCCT-3′ |
| *LIPT1*: sense | 5′-CAGGAACAGCTTCTAAGATCGG-3′ |
| *LIPT1*: antisense | 5′-ACAAGAACGTCCCATCAGTAC-3′ |
| *MTF1:*sense | 5′-GTAATGACCTTCCCATATCCCC-3′ |
| *MTF1:*antisense | 5′-GCTCCTATTTCCCACTTCCG-3′ |
| *PDHA:*sense | 5′-AGCCGCAACCAGTCTATAAG-3′ |
| *PDHA:*antisense | 5′-CAGGTATTACAGAGAACCCAGC-3′ |
| *PDHB:*sense | 5′-CGGTGTCTGGCTTGGTG-3′ |
| *PDHB:*antisense | 5′-CCTTCTCATCTCTTTCCAGCTC-3′ |
| *β-actin:*sense | 5′-ACACTGTGCCCATCTACGAG-3′ |
| *β-actin*antisense | 5′-TCAACGTCACACTTCATGATG-3′ |
