# Supplementary material for: Molecular subtypes, tumor microenvironment infiltration characterization and prognosis model based on cuproptosis in bladder cancer
Source: PeerJ. 2023 Apr 6;11:e15088. doi: 10.7717/peerj.15088 (PMC10083007; doi:10.7717/peerj.15088)
Supplement: Supplemental Information 3 [file peerj-11-15088-s003.docx]

**Supplementary Table S3 Univariate Cox regression and Kaplan–Meier analysis of 71 cuproptosis related genes in TCGA**

| Symbol | KM_Pvalue | Cox_Pvalue | HR | HR.95L | HR.95H |
| --- | --- | --- | --- | --- | --- |
| SYDE1 | 0.018327504 | 0.006997233 | 1.04952131 | 1.013296061 | 1.087041608 |
| RBP1 | 0.028765899 | 0.047158817 | 1.002358138 | 1.000029557 | 1.004692141 |
| THUMPD2 | 0.004332646 | 0.000835037 | 0.820266543 | 0.730257851 | 0.921369349 |
| COL6A3 | 0.015499541 | 0.00579653 | 1.004477951 | 1.001294891 | 1.007671129 |
| PDGFD | 0.010614595 | 0.001763567 | 1.072629934 | 1.026518466 | 1.12081274 |
| RPS6KA1 | 0.011724869 | 0.023713572 | 0.967939888 | 0.940989908 | 0.995661717 |
| TIMP2 | 0.019164934 | 0.03127393 | 1.002667236 | 1.000239494 | 1.005100871 |
| ZNF680 | 0.043684601 | 0.015195491 | 0.862663595 | 0.765673879 | 0.971939226 |
| RAB3IL1 | 0.003448752 | 0.012995617 | 1.047309312 | 1.009797756 | 1.086214332 |
| USP3 | 0.005579091 | 0.002825258 | 0.896604303 | 0.834622644 | 0.963188911 |
| TCF4 | 0.016023871 | 0.000166316 | 1.133323779 | 1.061845444 | 1.209613692 |
| PNMA1 | 0.049093384 | 0.016345314 | 1.015479329 | 1.002826165 | 1.028292145 |
| OGN | 0.000255127 | 0.033755191 | 1.036290117 | 1.002741028 | 1.070961671 |
| LCOR | 0.001449247 | 0.002423441 | 0.859442427 | 0.77929732 | 0.947829879 |
| COL6A2 | 0.010588946 | 0.009059427 | 1.000922876 | 1.000229716 | 1.001616516 |
| NES | 0.020849391 | 2.69E-05 | 1.02564982 | 1.013594083 | 1.037848949 |
| DPYSL2 | 0.007175685 | 0.002551783 | 1.02685411 | 1.00932849 | 1.044684038 |
| TNC | 0.023982336 | 0.035209236 | 1.004136191 | 1.000286162 | 1.008001038 |
| WDR45 | 0.008280535 | 0.00117599 | 0.934163459 | 0.896512726 | 0.973395404 |
| TGFB1I1 | 0.004903398 | 0.016015847 | 1.021883396 | 1.004039938 | 1.040043961 |
| ATP8B1 | 0.016259627 | 0.037105495 | 0.987427826 | 0.975751539 | 0.999243836 |
| BCS1L | 0.009912527 | 0.022263099 | 0.93425776 | 0.881340804 | 0.990351924 |
| GAS7 | 0.035109514 | 0.000274338 | 1.103818818 | 1.046620519 | 1.16414303 |
| ALDH1L2 | 0.000132301 | 7.54E-05 | 1.179596691 | 1.086970586 | 1.280115921 |
| ISCU | 0.001668647 | 0.032006703 | 0.970439984 | 0.944186439 | 0.997423521 |
| SETBP1 | 0.000556025 | 0.000239023 | 1.24917729 | 1.109365224 | 1.406609714 |
| TRIM11 | 0.043958846 | 0.041624437 | 0.940895059 | 0.887332112 | 0.997691282 |
| APCDD1L | 0.00617429 | 0.016145437 | 1.014645506 | 1.002697082 | 1.026736311 |
| KCNH4 | 0.03616226 | 0.002758226 | 0.547470017 | 0.369022477 | 0.812209117 |
| OSBPL10 | 0.005090984 | 0.001245687 | 1.135197768 | 1.051079501 | 1.22604805 |
| AGTR1 | 0.006692606 | 0.039477889 | 1.101520665 | 1.004669277 | 1.20770865 |
| MAPRE2 | 0.004014592 | 0.024563905 | 1.037570679 | 1.004739652 | 1.071474498 |
| SERPINE2 | 0.003137487 | 0.023236129 | 1.016500421 | 1.002234798 | 1.030969098 |
| NTNG1 | 0.011565736 | 0.00509456 | 1.33741288 | 1.091220745 | 1.639148835 |
| CSPG4 | 0.003659265 | 0.002502537 | 1.016931621 | 1.005921739 | 1.028062007 |
| COL8A1 | 0.030529074 | 0.002764987 | 1.028583474 | 1.009772991 | 1.047744367 |
| ZNF721 | 0.02268596 | 0.016378564 | 0.856584423 | 0.754875371 | 0.971997367 |
| COPZ2 | 0.039269615 | 0.000435456 | 1.024968989 | 1.010980671 | 1.039150854 |
| TGFB3 | 0.007160151 | 0.008269766 | 1.030636014 | 1.007810623 | 1.053978366 |
| CHMP4C | 0.000845827 | 0.004386266 | 0.973198474 | 0.955178392 | 0.991558517 |
| MAP1A | 3.63E-05 | 0.004123909 | 1.093763109 | 1.028793811 | 1.162835279 |
| SFRP2 | 0.047565979 | 0.037811882 | 1.001006634 | 1.000056644 | 1.001957527 |
| CTPS2 | 0.007466476 | 0.005505922 | 0.901680482 | 0.838139446 | 0.970038692 |
| CTGF | 0.011913603 | 0.038839046 | 1.001639935 | 1.000084019 | 1.003198271 |
| KCNJ8 | 0.031998362 | 0.018312528 | 1.053319073 | 1.008830048 | 1.099770047 |
| EPHA3 | 0.010677496 | 0.001655671 | 1.097290271 | 1.035620109 | 1.16263283 |
| CPE | 0.003660854 | 0.028340295 | 1.005973181 | 1.000631934 | 1.011342938 |
| CD248 | 0.043432514 | 0.018032998 | 1.003387717 | 1.000579305 | 1.006204012 |
| HSPG2 | 0.001089022 | 0.003241699 | 1.020114741 | 1.006678123 | 1.033730705 |
| FASTKD1 | 0.004832236 | 0.008921737 | 0.876961841 | 0.794776269 | 0.967645992 |
| TNFAIP8L3 | 0.003353575 | 0.001201532 | 1.072123222 | 1.027875412 | 1.118275804 |
| MYADM | 0.028984621 | 0.004220622 | 1.007181047 | 1.002256356 | 1.012129936 |
| PLSCR4 | 0.018948001 | 0.026519202 | 1.083208027 | 1.009358658 | 1.162460558 |
| PIGA | 0.027614827 | 0.00379439 | 0.920425849 | 0.870175125 | 0.973578442 |
| MMAA | 0.007276017 | 0.022380893 | 0.683603133 | 0.493213449 | 0.947486823 |
| GSN | 0.001422077 | 0.003756443 | 1.006440235 | 1.00207996 | 1.010819483 |
| ATP8B2 | 0.007843149 | 0.004044537 | 1.028524348 | 1.008989416 | 1.048437493 |
| ZNF700 | 0.003916028 | 0.024058313 | 0.927501679 | 0.868802182 | 0.990167131 |
| CCDC80 | 0.006161878 | 0.002353902 | 1.012977083 | 1.004595444 | 1.021428652 |
| ACP6 | 0.006545862 | 0.002341079 | 0.91431527 | 0.863057278 | 0.968617535 |
| CLIC4 | 0.014375831 | 0.024591056 | 1.005690621 | 1.000726823 | 1.010679041 |
| SIRT5 | 0.040699349 | 0.007277562 | 0.858675105 | 0.768255562 | 0.959736542 |
| PID1 | 0.000111448 | 0.011105572 | 1.069126225 | 1.01536789 | 1.125730778 |
| DPYSL3 | 0.030248791 | 0.021633765 | 1.005696384 | 1.000833244 | 1.010583155 |
| HARS2 | 0.034332495 | 0.041544181 | 0.959310594 | 0.921742814 | 0.998409537 |
| GANAB | 0.014550206 | 0.001485037 | 1.006514711 | 1.002491387 | 1.010554182 |
| NFIA | 0.00217777 | 0.020220121 | 1.047989254 | 1.00733914 | 1.090279762 |
| GNB4 | 0.033247855 | 0.027168112 | 1.05611038 | 1.006175667 | 1.108523265 |
| FAM20C | 0.039241318 | 0.001498863 | 1.023595972 | 1.008964525 | 1.038439596 |
| LGALS1 | 0.031222174 | 0.000633101 | 1.000609228 | 1.000259737 | 1.000958842 |
| RBMS3 | 0.003982818 | 0.015756893 | 1.230933373 | 1.039888662 | 1.45707615 |
