# Supplementary material for: Molecular subtypes, tumor microenvironment infiltration characterization and prognosis model based on cuproptosis in bladder cancer
Source: PeerJ. 2023 Apr 6;11:e15088. doi: 10.7717/peerj.15088 (PMC10083007; doi:10.7717/peerj.15088)
Supplement: Supplemental Information 4 [file peerj-11-15088-s004.docx]

**Supplementary Table S4 The relative infiltration levels of TIICs based on the risk groups.**

| Type of immune cell | Low risk group | High risk group |
| --- | --- | --- |
| B cells naive | 0.039465854 | 0.06603841 |
| B cells memory | 0.023816562 | 0.008440292 |
| Plasma cells | 0.031612484 | 0.031395303 |
| T cells CD8 | 0.139762992 | 0.10852297 |
| T cells CD4 naive | 0.008582277 | 0.002589265 |
| T cells CD4 memory resting | 0.092182891 | 0.094095651 |
| T cells CD4 memory activated | 0.042967204 | 0.050391068 |
| T cells follicular helper | 0.045815936 | 0.031436347 |
| T cells regulatory (Tregs) | 0.026787446 | 0.02160625 |
| T cells gamma delta | 0.006294754 | 0.002373226 |
| NK cells resting | 0.018169285 | 0.012759005 |
| NK cells activated | 0.030714422 | 0.022824462 |
| Monocytes | 0.015651214 | 0.011025062 |
| Macrophages M0 | 0.095047225 | 0.166991364 |
| Macrophages M1 | 0.052311416 | 0.073977789 |
| Macrophages M2 | 0.117621914 | 0.148838472 |
| Dendritic cells resting | 0.054362426 | 0.031259875 |
| Dendritic cells activated | 0.077559098 | 0.037973787 |
| Mast cells resting | 0.054762177 | 0.051693723 |
| Mast cells activated | 0.015411796 | 0.013069727 |
| Eosinophils | 0.002909413 | 0.002399061 |
| Neutrophils | 0.008191213 | 0.010298891 |
