# Supplementary figures and images for: Molecular subtypes, tumor microenvironment infiltration characterization and prognosis model based on cuproptosis in bladder cancer
Source: PeerJ. 2023 Apr 6;11:e15088. doi: 10.7717/peerj.15088 (PMC10083007; doi:10.7717/peerj.15088)

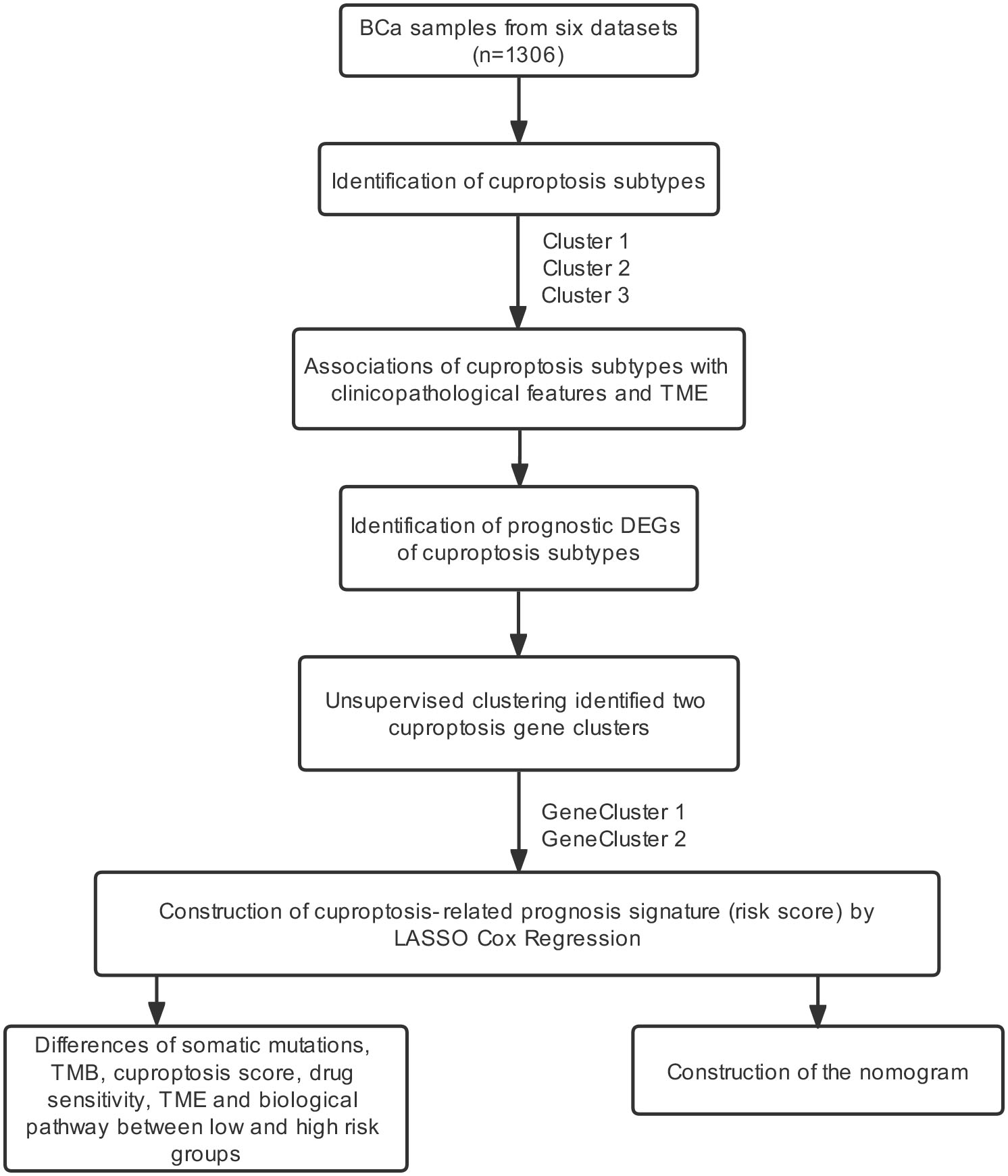

Supplement: Supplemental Information 5 [file peerj-11-15088-s005.png]

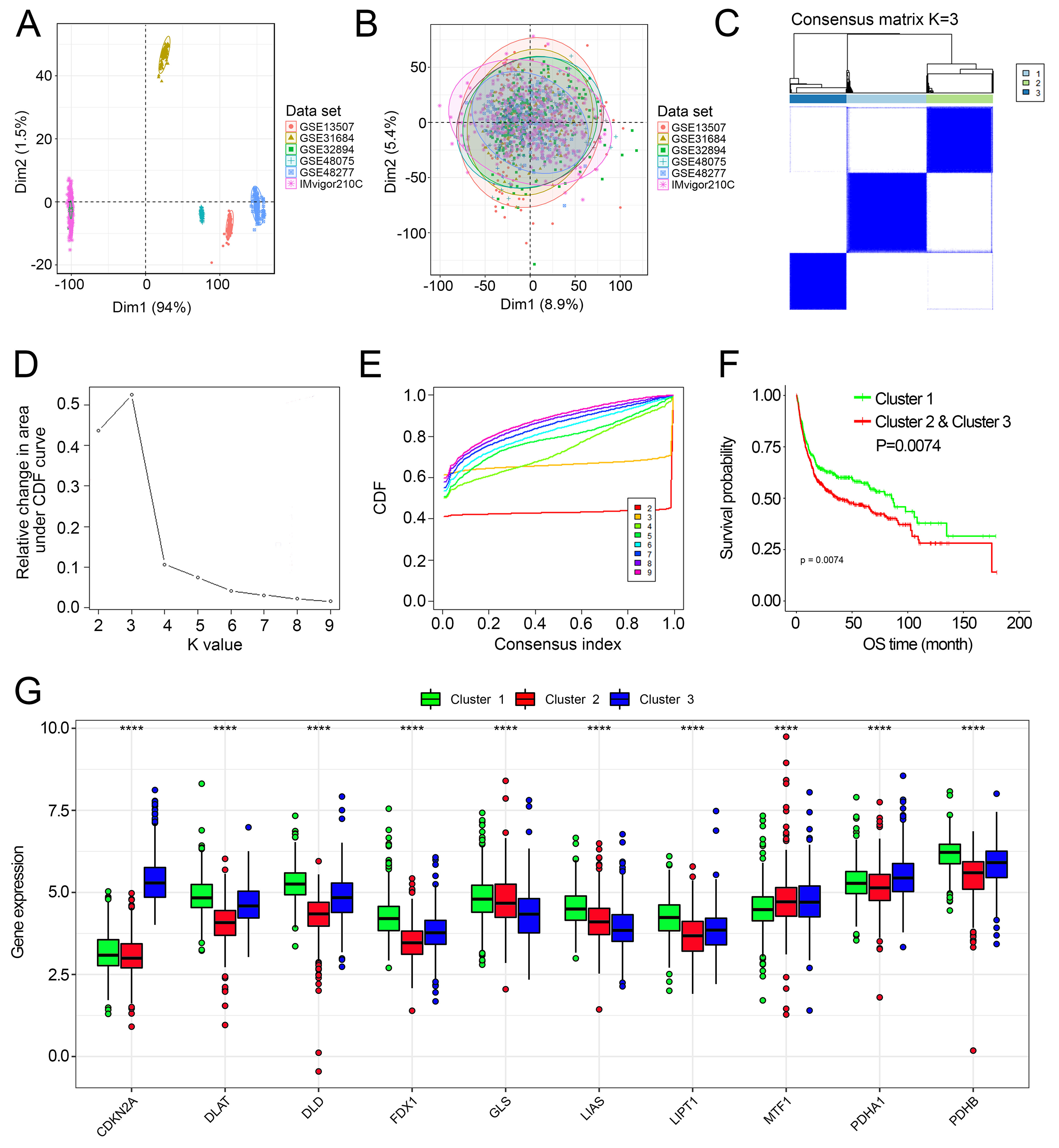

Supplement: Supplemental Information 6 — (A–B) PCA plots of the combined dataset before (A) and after (B) the elimination of the batch effects. c Consensus matrix heatmaps for k = 2. (D–E) Consensus clustering CDF (D) and relative change in area under CDF curve for k = 2–9 (E). (F) Kaplan–Meier curves for OS of the combined dataset with the cuproptosis-related subtypes. (G) The expression of cuproptosis-related genes among cuproptosis-related subtypes. [file peerj-11-15088-s006.png]

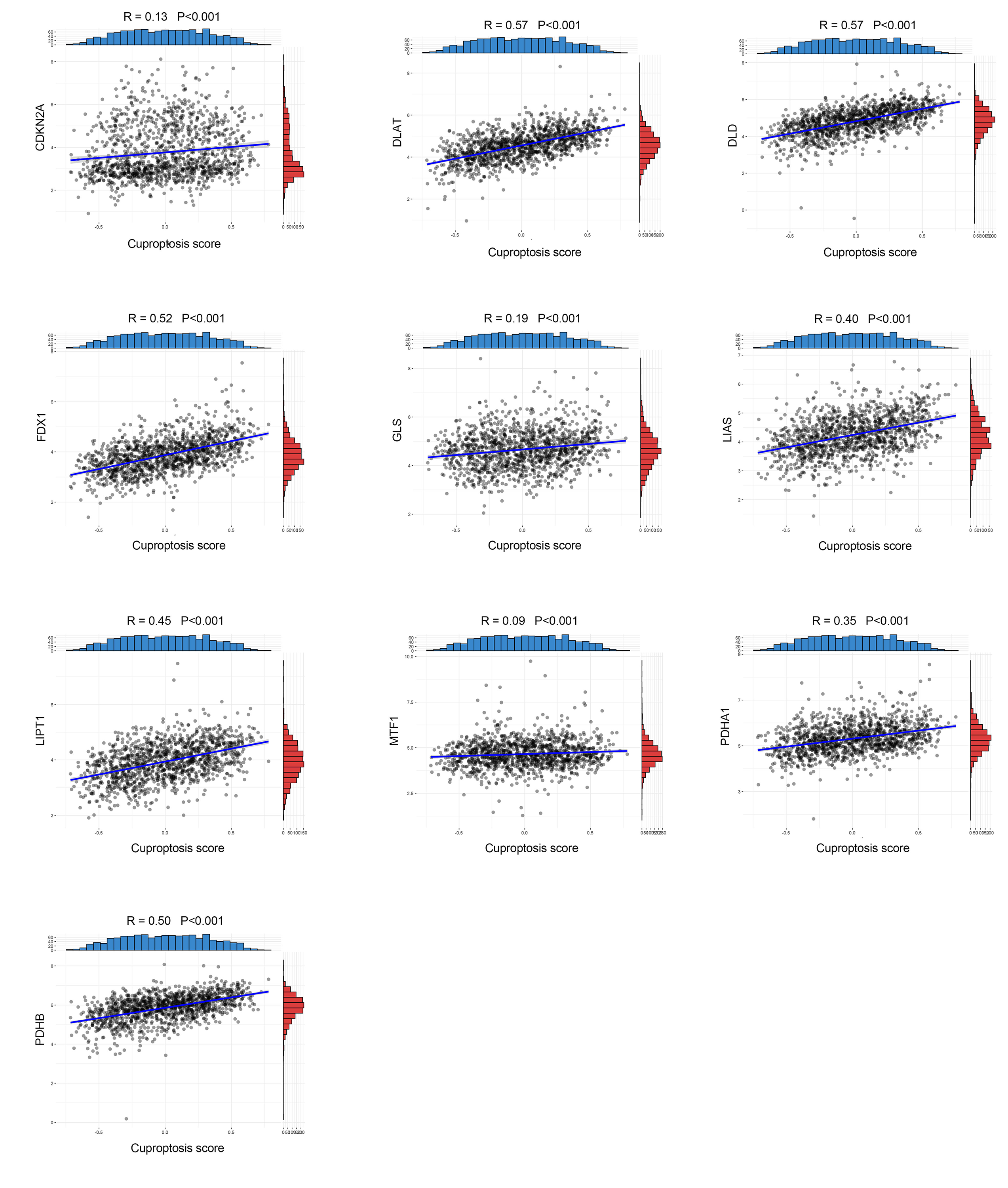

Supplement: Supplemental Information 7 [file peerj-11-15088-s007.png]

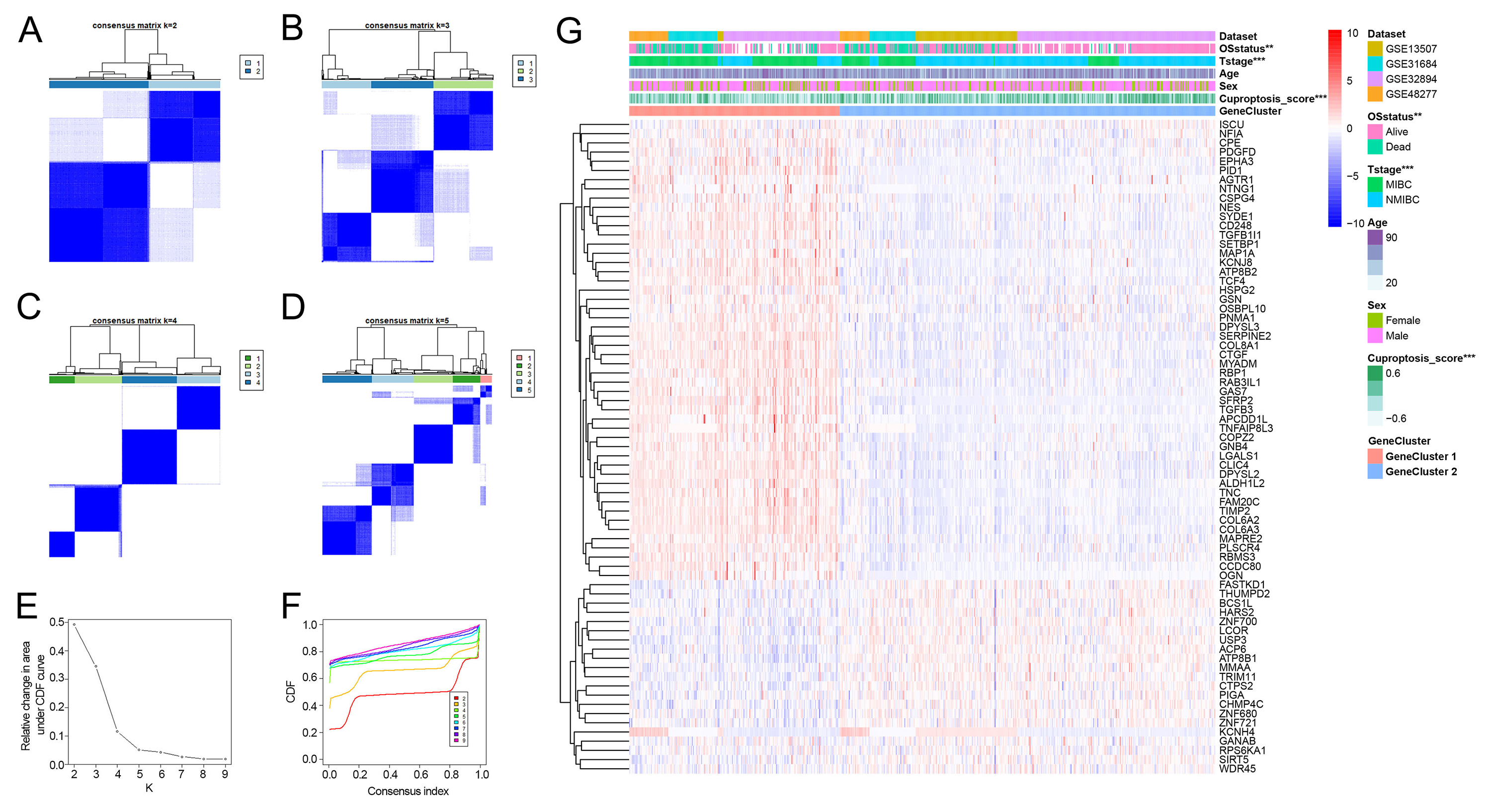

Supplement: Supplemental Information 8 — (A–D) Consensus matrix heatmaps for k = 2–5. (E–F) Consensus clustering CDF (E) and relative change in area under CDF curve for k = 2–9 (F). (G) Differences in clinicopathologic features between cuproptosis gene clusters. [file peerj-11-15088-s008.png]

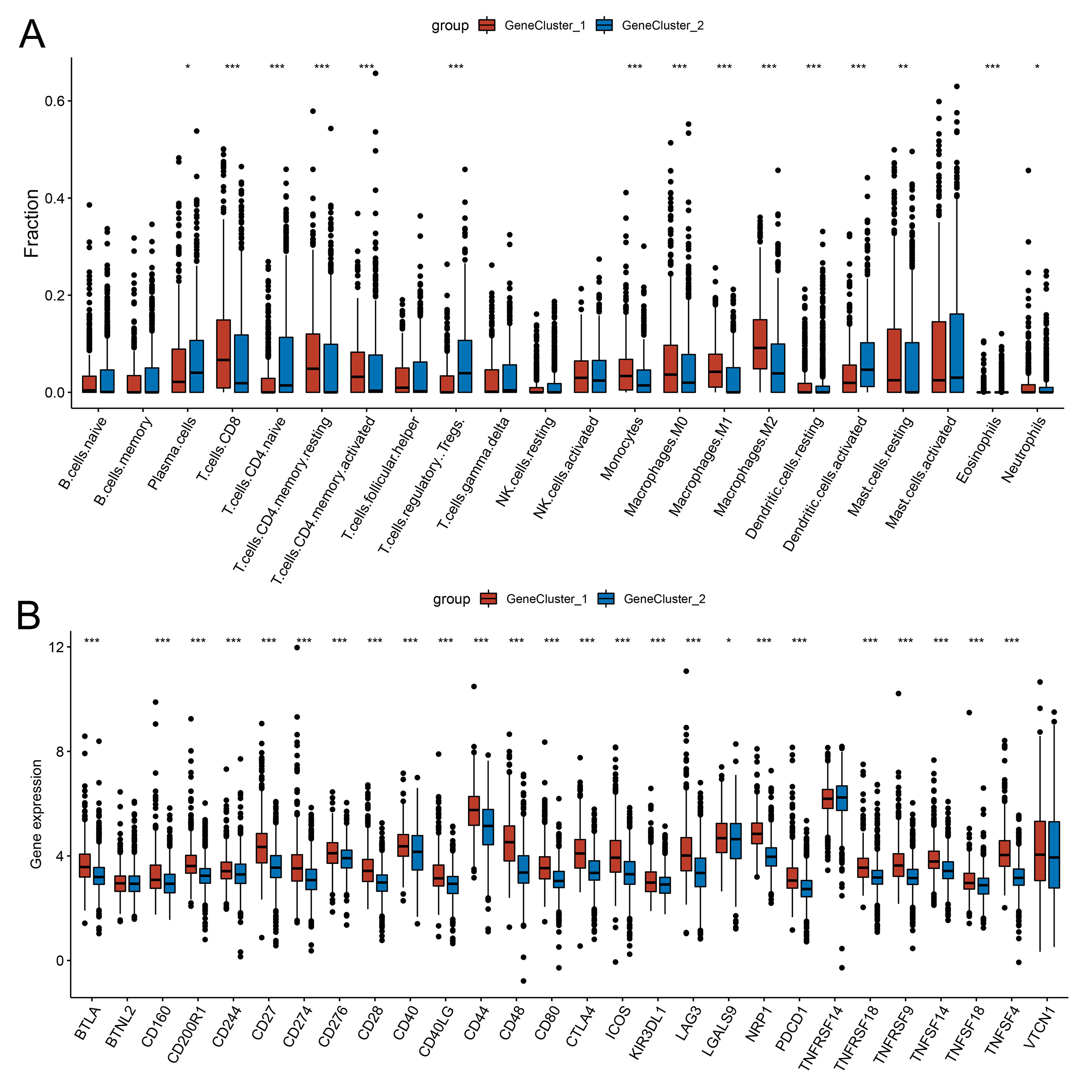

Supplement: Supplemental Information 9 — (A) The relative infiltration levels of TIICs based on the cuproptosis gene clusters. (B) The expression of immune checkpoints based on the cuproptosis gene clusters. [file peerj-11-15088-s009.png]

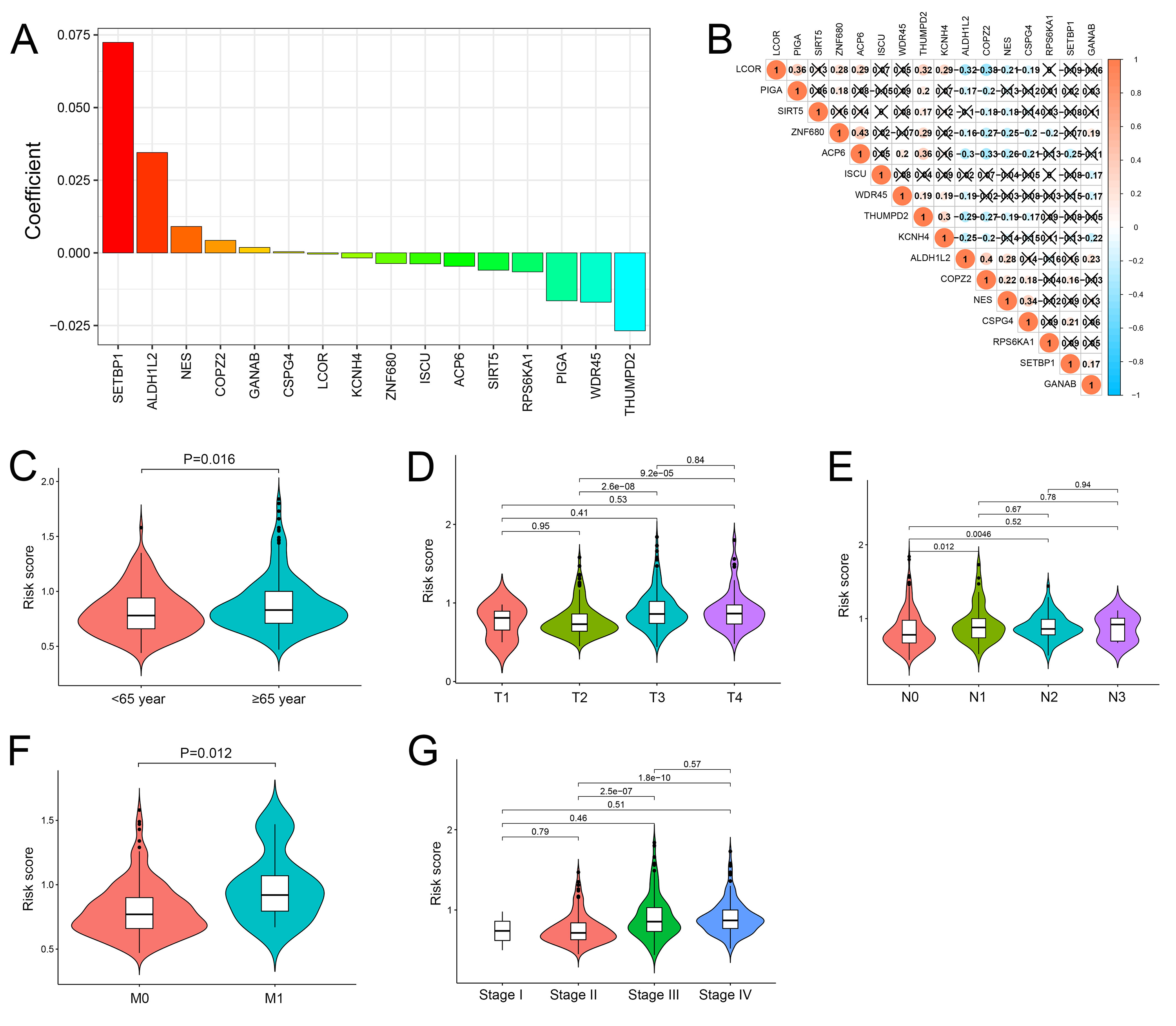

Supplement: Supplemental Information 10 — (A) Coefficients of 16 genes in the cuproptosis-related prognosis signature. (B) Correlations among 16 genes in the cuproptosis-related prognosis signature. (C–G) Distribution of risk scores stratified by age (C), T stage (D), N stage (E), M stage (F) and Stage (G). [file peerj-11-15088-s010.png]

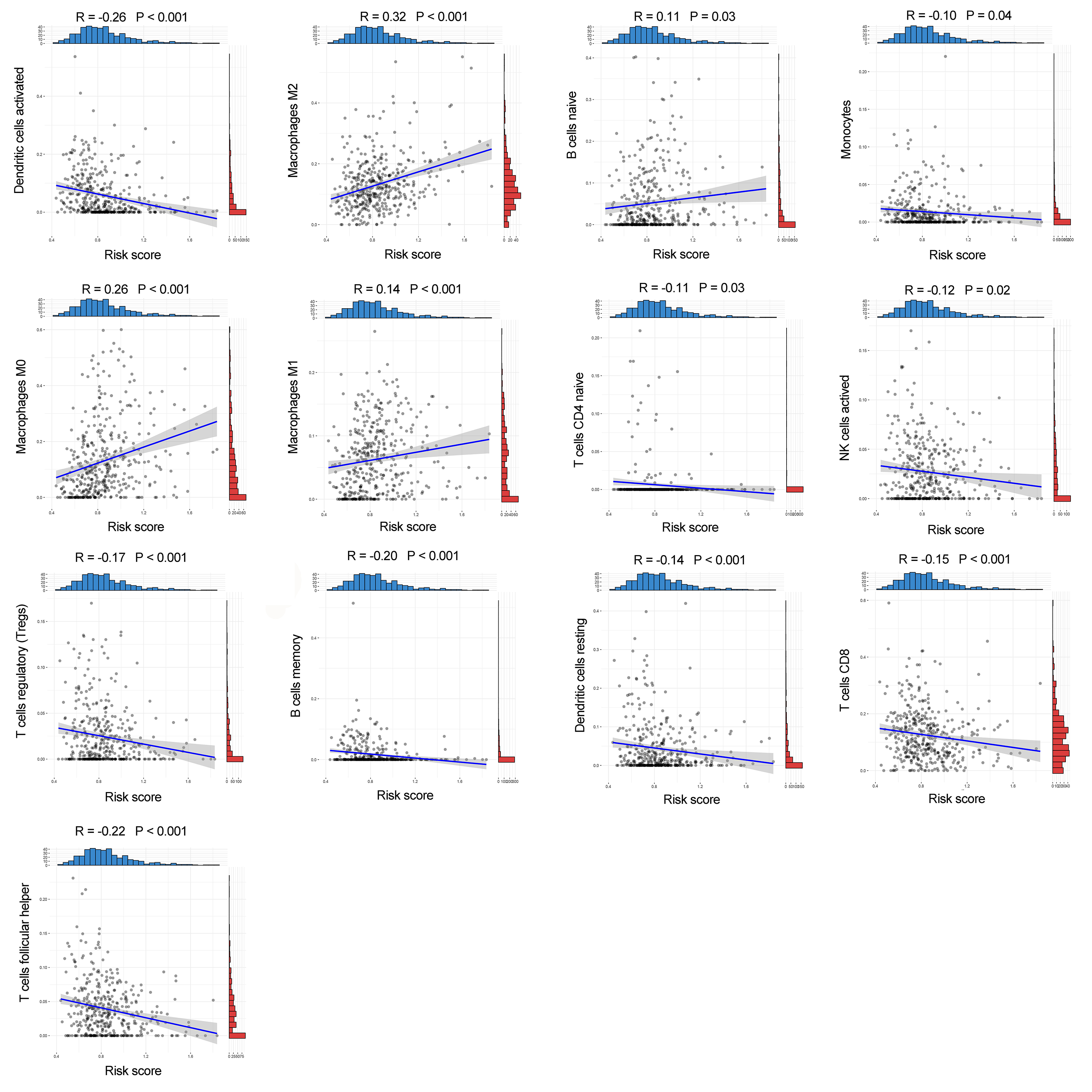

Supplement: Supplemental Information 11 — The correlation between the cuproptosis-related prognosis signature (risk score) and tumor-infiltrating immune cells. [file peerj-11-15088-s011.png]
